# Nanobubbles in Bulk Solution and Electrosprayed Microdroplets Accelerate Chemical Reactions

**DOI:** 10.1021/jasms.6c00359

**Published:** 2026-09-09

**Authors:** Bincy Binny, George Joseph, Andre R. Venter

**Affiliations:** 4175 Western Michigan University, Kalamazoo, Michigan 49008-5413, United States

## Abstract

Bulk nanobubbles (NBs) are metastable gas-filled voids in solution, with diameters of nearly 200 nm that exhibit long lifetimes and large surface-to-volume ratios. Recent studies have shown that chemical reactions are significantly accelerated within sprayed microdroplets during electrospray ionization mass spectrometry (ESI-MS). Although several factors, such as partial solvation of reagents, electric fields, ordered orientation, and pH, have been implicated in the acceleration in microdroplets, the primary governing factor remains unclear and dependent on the system. Similar processes occur on the nanobubble–liquid interface, leading to enhanced reaction rates when they are present in bulk solution. We demonstrate reaction rate acceleration by deliberately adding NBs to the bulk solution and microdroplets by considering two model systems: the Katritzky transamination reaction and the reaction between CO_2_ and *N*,*N*-dibutyl-1,3-propane diamine (DBPA). N_2_ and CO_2_ nanobubbles were generated using sonication, pressure cycling (PC), or the Tesla valve (TV) and delivered as bulk or spray solvents. The Katritzky reaction in bulk solution with NB-enriched solvent systems was examined by mass spectrometry under nonaccelerating electrospray conditions. The acceleration factor (AF) for the reaction doubled compared with the control. On the other hand, the microdroplet accelerated the Katritzky reaction, with N_2_ NB producing a further six-fold acceleration compared to the microdroplet control. For the reaction of the diamine DBPA with N_2_ nebulization, CO_2_ reagent delivery by NB resulted in acceleration factors between 2 and 50 compared to the microdroplet reaction control, depending on the solvent fraction, wherein bubbles were generated. With CO_2_, nebulization where ample reagent is already present, the addition of NBs produced a further two-to-five-fold acceleration factor depending on the solvent fraction in which NBs were generated, indicating a NB catalytic effect. Our findings show that NBs improve reaction kinetics by acting both as improved reagent delivery vehicles and/or as catalysts. Catalytic activity is a consequence of increased liquid–air interfacial surface area and improved reagent transport in bulk solution and within microdroplets.

## Graphical abstract

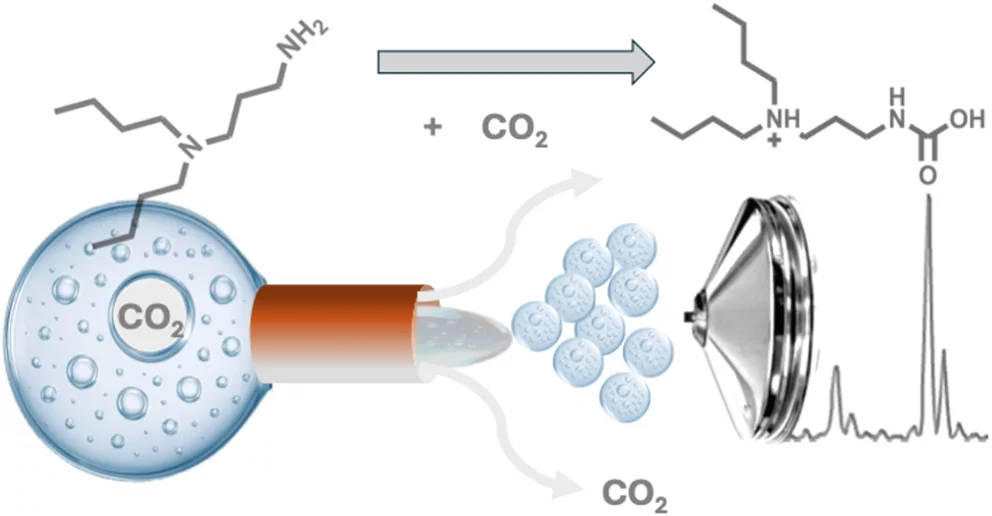

## Introduction

Electrospray ionization mass spectrometry (ESI-MS) is a versatile analytical technique widely employed for the analysis of small molecules,^1^ proteins,^2^ and lipids.^3^ Beyond its conventional application, ESI-MS has proven to be an invaluable tool for real-time monitoring of chemical reactions, allowing the detection of reaction species, intermediates, and products.^4–6^ Lately, microdroplet reaction chemistry has been of keen interest. While initially explored for monitoring chemical reactions,^6,7^ studies have shown that reactions can be significantly accelerated in microdroplets produced during ESI-MS, with reactions occurring in milliseconds to microseconds timeframe during their flight to the MS.^8–12^ The acceleration of reactions by microdroplets is important, as it can potentially make reactions feasible that are too slow in bulk solution and allow for high-throughput reaction screening.^13,14^ Accelerated reactions during electrospray could also lead to the production of “phantom” constituents during the analysis of complex mixtures, leading to the detection of compounds not natively present in samples. Research by the Cooks and Zare groups has demonstrated that microdroplet-mediated reactions, such as redox processes, acid–base catalysis, and enzyme digestion, can proceed up to 5 orders of magnitude faster than their bulk counterparts.^11,15^ An increasing number of studies, including several recent reviews, summarize the current understanding of reaction acceleration in microdroplet environments.^8,12,15–17^ Acceleration of the reaction in microdroplets over the bulk is attributed to the unique liquid–air interfacial environment. Reagent confinement,^15,17^ partial solvation of reactants at interface,^18^ reagent concentration due to solvent evaporation, pH changes,^9^ increased surface-to-volume ratios of microdroplets,^19^ and reagent alignment associated with large electric fields at the air droplet interface^20^ are all factors associated with the acceleration of reactions in microdroplets. The accelerated reaction rate in microdroplets is likely due to several factors rather than a single parameter, although some factors may contribute more substantially. For example, in the mechanistic investigation of the aza-Michael addition in methanol microdroplets, the surface acidity was identified as a predominant accelerating factor, lowering the activation energy barrier by approximately 9 kcal/mol. In contrast, interfacial electric fields contributed only modestly (∼2 kcal/mol), while partial solvation had a negligible effect on acceleration.^21^ Narendra et al. demonstrated by quantum mechanical modeling that solvation energy differences between the bulk phase and the gas–liquid interface of microdroplets can substantially change the activation energies of reactants and transition states in microdroplets, highlighting differential solvation as a dominant factor in rate acceleration in the hydrazone formation reaction.^22^ The reactions of amines with CO_2_ to yield carbamic acids were significantly accelerated in the presence of trace amounts of water, where the superacidic/superbasic environment on the droplet surface due to H_2_O was speculated as the major contributor for the acceleration.^12^

Water exhibits unique properties at the gas–water interface of microdroplets. At the interface, symmetry is disrupted and charge separations give rise to a strong interfacial electric field. The presence of strong electric fields (10^7^–10^9^ V/m) on the gas–liquid interface of microdroplets has been demonstrated using stimulated Raman excited fluorescence microscopy.^20,23^ The electric field is reported to originate from the formation of an electric double layer on the surface of the droplet,^24,25^ alignment of free O–H bonds of interfacial water,^23^ or partial charge transfer resulting in interfacial H_2_O^+^–H_2_O^–^ pairs.^20,23,24,26–29^ The electric field present at the microdroplet interface has been proposed to accelerate microdroplet reactions or even induce spontaneous reactions that are not favorable in bulk.^23,27,28^ The intrinsic electric field is large enough to pull electrons out of the hydroxide ions in water,^30–33^ to form hydroxyl radicals, which, in turn, induce other reactions. For instance, the Zare group demonstrated the spontaneous formation of H_2_O_2_ from water microdroplets, otherwise unfavorable in bulk.^30^ Numerous organic reactions conducted in water microdroplets have demonstrated substantial acceleration relative to their bulk counterparts.^27–29,34^ For example, the Menshutkin reaction was experimentally accelerated by 7 orders of magnitude, and DFT and *ab initio* molecular dynamics simulations predicted that the reaction barrier decreases significantly in the presence of the oriented external electric field.^27^

Nanobubbles (NBs) are metastable gas-filled voids in solution with diameter distributions in the tens to hundreds of nanometers, dependent on gas and solvent properties. They exhibit unique chemical and physical properties, for example, enhanced mass transfer efficiency by their negligible buoyancy, longevity, and slow dissolution into liquid phases.^35,36^ They are metastable in solution due to reasons that are still actively being investigated. Hypotheses include electrostatic effects preventing complete collapse of the bubble,^37,38^ polarization effects,^39^ the existence of a dielectrostatic force,^40^ thermodynamically due to the energy requirement of gas dissolution,^41^ etc.

They remain long-lived in solution because of their electrical double layer (EDL) and do not coalesce to grow to the point where they have adequate buoyancy to rise and burst at the liquid surface, but can remain stable in solution for several days to weeks.^42^ They have a strong affinity for hydrophobic surfaces, the ability to generate free radicals,^43–45^ possess high internal pressures due to surface tension (30 atm for a 100 nm bubble in water), and have very high surface-to-volume ratios, which enhance their gas-loading capacities. Similar to microdroplet surfaces, NB interfaces become charged due to the adsorption of OH^–^ ions and interfacial forces orient water molecules at the first and subsequent layers.^46^ Thus, similar to microdroplets, they possess strong electric fields (∼10^6^ to 10^8^ V/m).^40^ The electric field in the Stern Layer contains a gradient component due to exponential decays with distance away from the interface.^40^ This electric field gradient exerts force on the dipole moments of oriented water (and likely reagent) molecules in the first monolayer and beyond.^40,47^ The unique properties of NBs have enabled their applications in various fields such as wastewater treatment,^48–50^ pesticide removal,^51^ solid surface cleaning,^52^ and other applications.^35,53^

The creation or existence of bubbles during ESI has been hypothesized to explain various observations, such as electrothermal supercharging,^54,55^ increased reaction rate of amines with carbon dioxide microbubbles produced from ammonium bicarbonate during reactive ESI,^56^ and improved desorption efficiency of proteins by desorption electrospray ionization (DESI-MS) when ammonium bicarbonate is used as an additive.^57^ The microbubbles in these studies were generated *in situ*.

Like microdroplets, NBs provide large gas–liquid interfaces where spatial confinement, partial solvation, pH changes, and reagent alignment associated with large electric fields at the interface could occur. A recent study from our group demonstrated that the deliberate addition of NBs into spray solutions in electrospray ionization enhanced the signal response of small molecules in both positive- and negative-ionization modes.^58^ Additionally, incorporating NBs into spray solutions during protein analysis by ESI increased charge states, confirming the role of ammonium bicarbonate-generated bubble formation during electrothermal supercharging.^55^

Beyond the mass spectrometry applications of NBs as noted above, bubbles were shown to enhance chemical reaction rates. For instance, O_2_ fine bubbles (<100 μm) were shown to enhance the oxidation of benzaldehyde to benzoic acid employing the slow rising velocity and increased surface area of micrometer-sized bubbles to increase the efficiency of O_2_ consumption.^59^ NB addition to an HBr etching solution was demonstrated to accelerate the etching process of gold nanorods compared to standard conditions with only dissolved oxygen.^36^ Furthermore, the photocatalytic degradation of methylene blue dye was enhanced in the presence of NBs.^60^ A recent study proposed that spontaneous electrical discharge, known as microlightning, occurring between interacting microbubbles, may explain the mysterious will-o′-the-wisp phenomenon.^61^

These findings demonstrate the potential of bubbles to influence chemical processes and have prompted us to explore the use of NBs to accelerate chemical reactions in bulk solutions, as well as electrosprayed microdroplets. The unique interfacial properties of NBs, particularly their large surface-to-volume ratio and interfacial environment, may be leveraged by their deliberate addition in both bulk solution and microdroplet environments.

Unlike previous studies that focused on specific bulk-phase systems involving the delivery of reactive gases, primarily O_2_ and O_3_, the present work investigates the catalytic effects of the interface in addition to, and in contrast to, reagent delivery.

The potential of NBs for the acceleration of bulk solution and microdroplets reaction rates using NBs is demonstrated using two model reactions, the Katritzky reaction and the reaction of diamine with CO_2._ During the Katritzky reaction, N-substituted pyridinium cations are formed from functionalized amines and pyrilium ions (Scheme 1).^9^ Here, the NBs do not deliver reagents but only serve as a reaction catalyst. The CO_2_ sequestration with amines (Scheme 2) was also conducted in both bulk and microdroplets, and the nanobubbles were used to both deliver reagents and act as a catalyst or simply to act as a catalyst.^12^

**Scheme 1:**
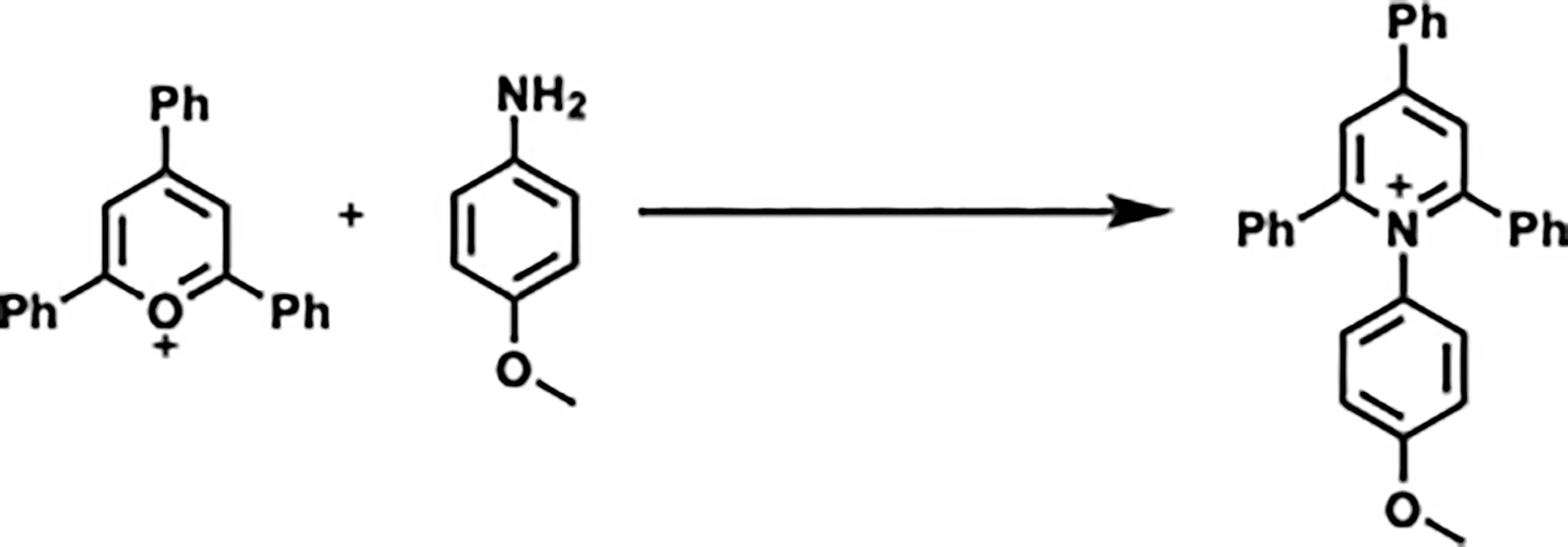
Reaction Scheme for the Katritzky Reaction between Pyrylium Ion (2,4,6-Triphenylpyrylium Tetrafluoroborate (TPP)) with Functionalized Amine (*p*-Anisidine) Forming the Substituted Pyridinium Cation

**Scheme 2:**
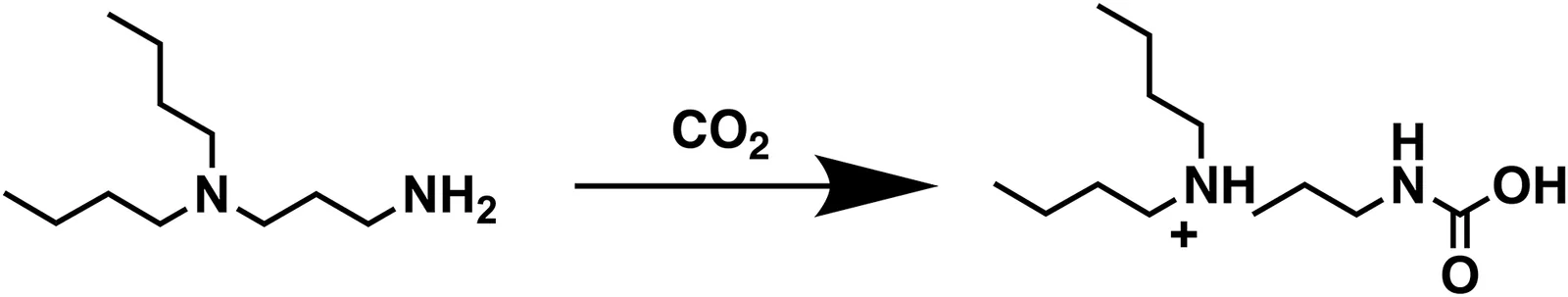
Reaction of DBPA with CO_2_, Forming the Product Carbamic Acid in the Positive Mode of Ionization

## Experimental Section

### Materials and Methods

2,4,6-Triphenylpyrilium tetrafluoroborate (TPP), *p*-anisidine, and *N*,*N*-dibutyl-1,3-propane diamine (DBPA) were purchased from Sigma-Aldrich (St. Louis, MO). Milli-Q water (18 MΩ cm^–3^) was obtained from a Thermo-Barnstead water polisher (Thermo Scientific, Waltham, MA, USA). A 99% gradient grade of HPLC acetonitrile (ACN) and HPLC grade ethanol was purchased from Sigma-Aldrich (St. Louis, MO).

### Nanobubble Generation

A 3% aqueous ethanol solution or pure acetonitrile (ACN) was sparged with >99% CO_2_ purchased from Airgas (PA, USA) for 5 min, delivered at 50 psi and a volumetric flow rate of 4.9 L/min. Similarly, a 3% ethanol solution or ACN was sparged with 99.999% N_2_ (Airgas, Gwinnett, Georgia) for 5 min, delivered at 50 psi and a volumetric flow rate of 7.1 L/min. These gas-saturated solutions were then used to generate NBs in water or ACN solvents.

NBs were generated using previously reported methods.^62–64^ A 3D-printed Tesla valve (TV) fabricated using a Phrozen Sonic Mini 8K LCD Resin 3D printer and Siraya Tech Blu resin (San Gabriel, California) was used to generate CO_2_ or N_2_ NBs by passing the carbonated or N_2_-sparged 3% ethanol solution through the valve for 12 forward–reverse flow cycles.^64^ NBs were also produced by a pressure cycling (PC) method, which involved repeatedly pulling and releasing the plunger of a 10 mL syringe containing the gas-sparged aqueous or acetonitrile solutions for an optimized number of 60 cycles.^63^ Sonication was performed using a Misonix Ultrasonic Liquid Processor XL-2020 probe (Misonix Farmingdale, NY) at 10 kHz for 5 min on gas-sparged aqueous or acetonitrile solutions.^62^ NB sizes and concentrations were determined by PMX-230 TWIN Laser Zeta View Nanoparticle Tracking Analysis system (NTA) (Particle Metrix, Ammersee, Germany). A detailed description of the protocols and optimization parameters for NB generation can be found in our previous publication.^64^ The sizes, concentration, and ζ-potential of N_2_ and CO_2_ NBs in water are detailed in Table S1. NBs in the ACN fraction could not be generated by the Tesla valve or characterized by NTA because the solvent was not compatible.

### Sample Preparation

#### Katritzky Reaction

Stock solutions of 5 mM TPP and *p*-anisidine were prepared in acetonitrile. The Katritzky reaction proceeded after 25 μL aliquots of both reagent stocks were combined in an Eppendorf tube and mixed by gentle inversion. Subsequently, the reaction was monitored periodically at predetermined time intervals by dilution of the resulting mixture 1000-fold with ACN immediately before analysis by ESI-MS under nonaccelerating conditions, where the ESI emitter was near the ion transfer capillary.^17^ Similarly, 5 mM stock solutions of TPP and *p*-anisidine were prepared in N_2_ NB-enriched ACN solutions, and the same analysis procedure was followed as described above.

#### CO_2_ Sequestration Reaction Using Diamine

For the CO_2_ sequestration reaction using amines, a 10 mM stock solution of DBPA was prepared in 100 % ACN and subsequently diluted to a final concentration of 10 μM in 95 % ACN: 5% H_2_O as previously optimized.^12^ For further details of optimization experiments of the reaction conditions, see the discussion in the Supporting Information and Figures S3–S5. N_2_ or CO_2_ NBs were generated in the ACN, water fraction, or both (total fraction). NBs in the total fraction were made either by generation in the separate solvents prior to mixing or by generation in the solvent premixture. CO_2_ and N_2_ NBs were generated in H_2_O or ACN using the three different techniques: pressure cycling (PC), flow regime switching using the Tesla valve (TV), or sonication (sonic). Unfortunately, the resins available to us for 3D printing were not compatible with ACN, and thus NBs could not be generated in some cases by the Tesla valve.

### Mass Spectrometry

A linear ion trap LTQ-XL mass spectrometer (Thermo Scientific, Waltham, MA) was combined with a 3-dimensional translational stage (Purdue University, West Lafayette, IN, USA) for analysis. Electrospray emitters were made from a Swagelok T-piece and two pieces of coaxial fused-silica capillary tubing. The outer capillary (for sheath gas) was approximately 20 mm in length, with an outer diameter of 360 μm and an inner diameter of 250 μm. The internal capillary (for solvent) had an outer diameter of 190 μm and an inner diameter of 75 μm. The solvent capillary extended through the T-piece and was connected to a syringe pump, which delivered the sample solution. The emitter was positioned 5.0 mm from the ion transfer tube at a 40° angle, and a 4 kV spray potential was applied to the liquid junction of the stainless steel syringe needle, which delivered the sample at a flow rate of 5 μL/min, with the nebulizing gas pressure set at 100 psi. The default nebulizing gas was nitrogen; however, CO_2_ was used in some cases as indicated. The capillary temperature was set at 250 °C. The capillary voltage and tube lens voltage were set at 20 and 65 V, respectively.

### Data Analysis

Mass spectra were collected and viewed in Xcalibur Qual Browser (2.0.7). Signal intensities were calculated as the average of three trials. Error bars represent ± mean standard deviation.

## Results and Discussion

The generation of NBs in the bulk solvent leads to an increase in the gas–liquid interfacial areas, where spatial confinement, partial solvation, pH changes, and reagent alignment associated with large electric fields at the bubble interface could occur. This should lead to an increase in reaction rates analogous to those achieved by microdroplet reactions, and for similar reasons, but in the bulk solution. Incorporating NBs into microdroplets has the potential to also synergistically amplify microdroplet effects, thereby further increasing the reaction rates. First, we investigated the effect of nanobubbles by way of the Katritzky reaction. Here, the bubble acts only as a catalytic surface, in the sense that it does not contain a reagent gas.

### Katritzky Reaction

The Katritzky reaction involves the conversion of the primary amines to pyridinium cations by reaction with pyrylium cations.^65^ Note that the published microdroplet studies concerning the reaction focus on the first step of the Katritzky amine displacement reaction, emphasizing the transformation of the amine rather than the ammonium salt (Scheme 1).^9,18,66,67^ The Cooks group first demonstrated that the reaction proceeded to completion when reagents were drop-cast onto a paper spray substrate and allowed the solvent to evaporate under ambient conditions for 10 min. The resulting mass spectra showed the m/z 414 product ion as a base peak and complete disappearance of the reagent ion at m/z 309.^9^ Further experiments of this reaction used an acceleration factor (AF) to express the reaction acceleration of Leidenfrost droplets compared to bulk solution.^66^ The acceleration factor was defined as the ratio of the MS product ion signal to the reagent ion signal in the Leidenfrost droplet versus the ratio of product to reagent ions in the bulk experiments. The ratio of ratios adjusts for inherent variations in ionization efficiencies.^66^ Leidenfrost droplets demonstrated an 8× acceleration factor compared to the bulk solution.^66^

The Katritzky reaction occurs slowly in the bulk solution (0.0010 min^–1^),^68^ allowing its use as a suitable model system to study the effect of NBs in bulk solution. Similar to the approach followed for the Leidenfrost experiments, we use the conversion ratio (CR) to define the ratio of the intensity of the product to the intensity of the reagent in any given system, while improvement in CR is the acceleration factor or the ratio of the ratios of the bubble system to control to account for differences in ionization efficiencies.

The bulk solution Katritzky reaction was performed as described in the Experimental Section, where the concentrated bulk reaction mixtures of both control and NB-containing solutions were periodically monitored by ESI under nonaccelerating or so-called monitoring conditions where the emitter is close to the ion transfer tube, and after 1000× dilution in pure ACN.^17^ The lack of acceleration associated with MS analysis was confirmed by the low product yield, barely reaching the limit of detection at time zero of control experiments without NBs (Figure S1). This setup allowed us to monitor the reaction without the complication of acceleration in the microdroplets that were subjected to MS analysis. As previously shown for other systems,^58^ addition of NBs in spray solutions increased signal intensities for both reactant and products over the studied intervals (Figure S2). Figure 1 shows the conversion ratio for the reaction in the control and NB system monitored over a period of 60 min. An increase in the conversion ratio over time as the reaction progressed in the bulk was observed for both NB-containing and control systems, as expected. Over 60 min, the conversion ratio of the control system rose to 7.9 × 10^–3^ while that of the NB system rose to 1.6 × 10^–2^, showing an acceleration factor of 2 for NB systems compared to controls.

**Figure 1:**
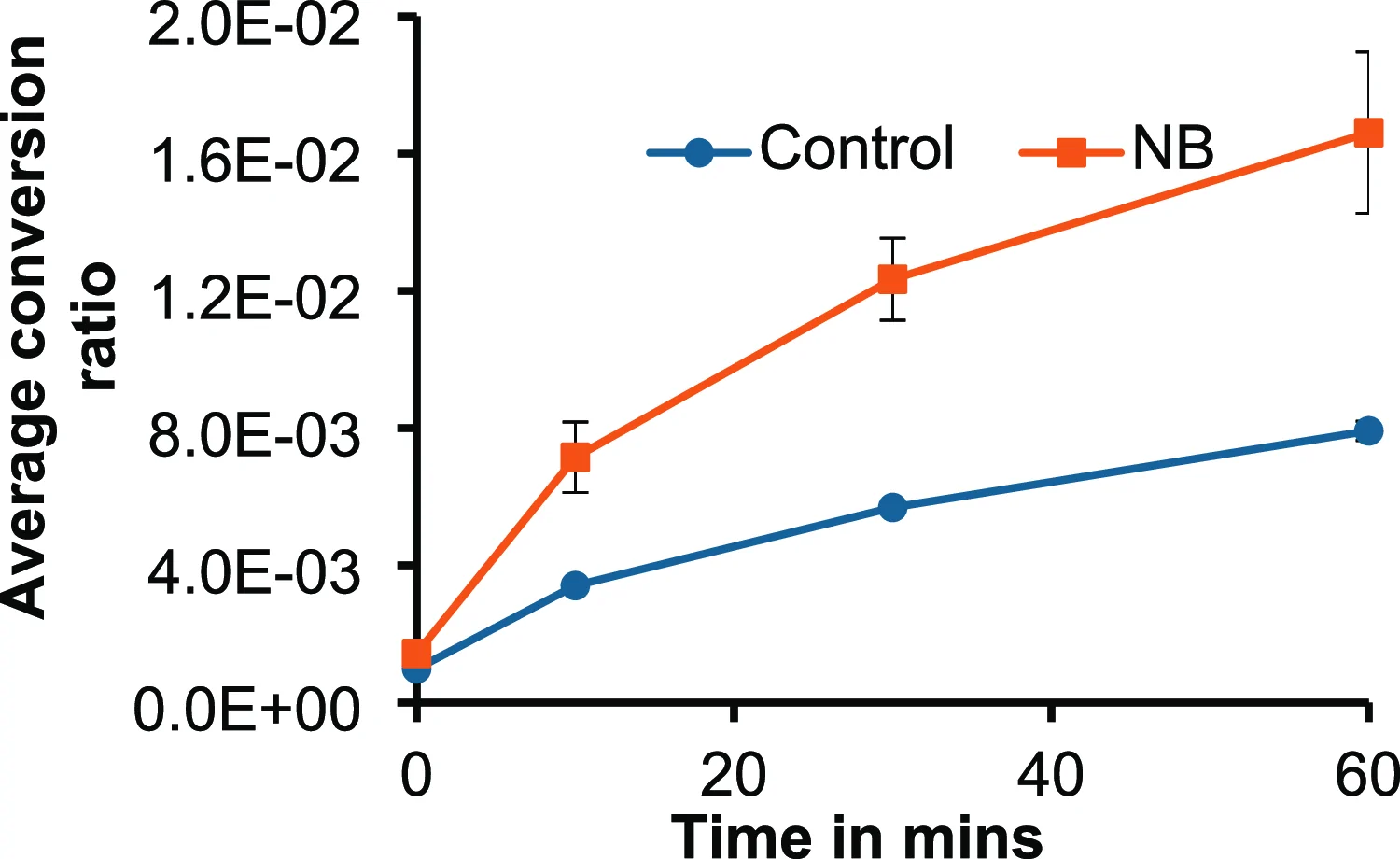
Conversion ratio of the reaction between 2,4,6-triphenylpyrylium tetrafluoroborate (TPP) and *p*-anisidine in acetonitrile in bulk at room temperature monitored over time by mass spectrometry. Results with N_2_ nanobubbles are shown in orange, and the control without NBs in blue.

In contrast, when the reaction mixture at time zero was diluted 1000× in N_2_ NB containing 100% ACN and monitored immediately, a 6× acceleration factor was obtained compared to the control, as shown in Figure 2. This acceleration factor is comparable to those obtained with Leidenfrost droplets.^66^

**Figure 2:**
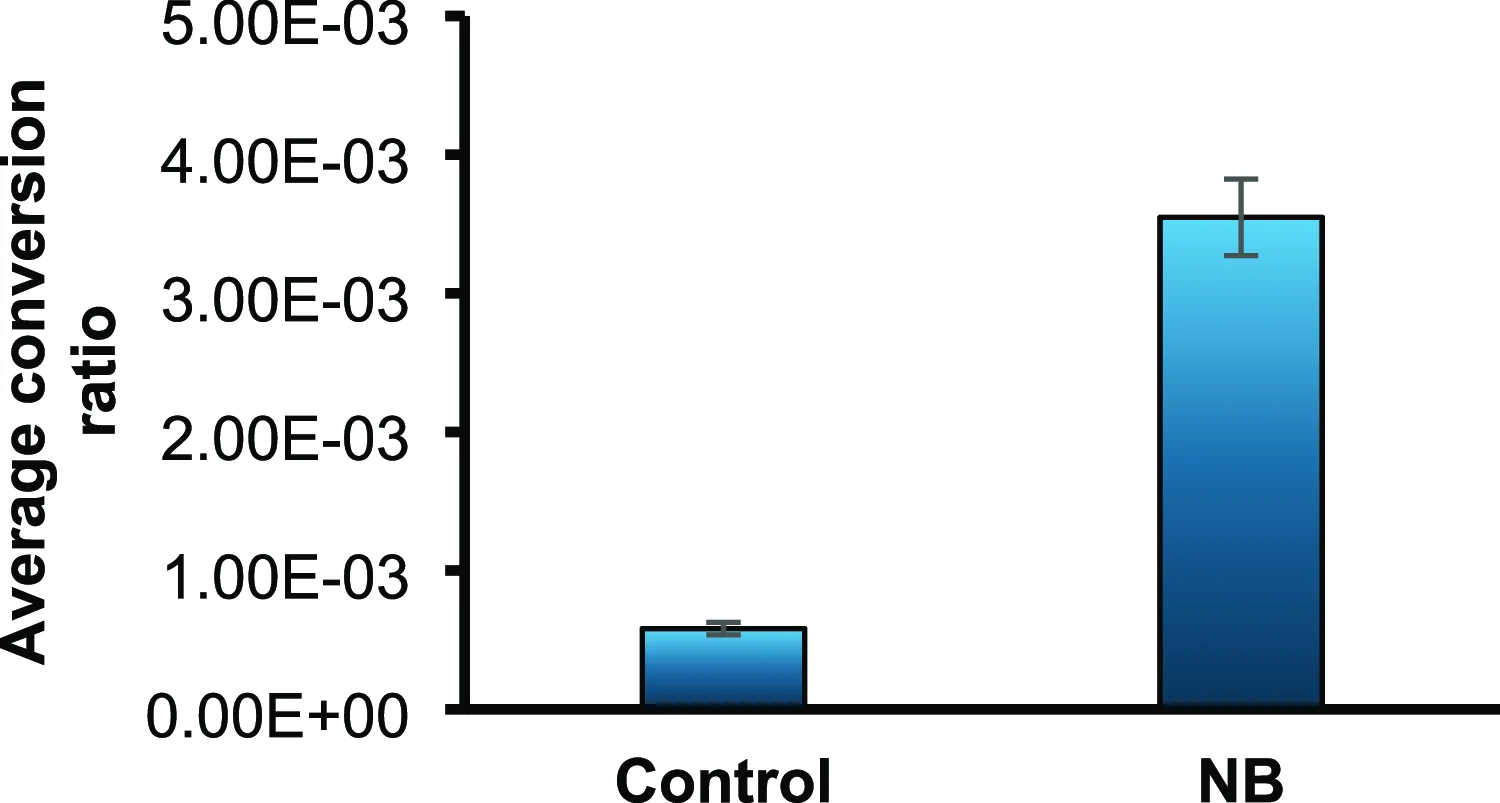
Katritzky microdroplet reaction with and without the N_2_ nanobubble addition.

### Reaction of DBPA with CO_2_

We also consider the NB acceleration of the CO_2_ sequestering reaction using the diamine, *N*,*N*-dibutyl-1,3-propane diamine (DBPA), illustrated in Scheme 2. Recently, the Cooks group demonstrated the deliberate addition of CO_2_ by electrosonic spray ionization (ESSI) using CO_2_ as nebulization gas. This enabled the gaseous CO_2_ to react with the amine only after the formation of microdroplets. Slow CO_2_ diffusion relative to droplet lifetime indicates that the reaction likely occurs only at the interface of the microdroplet. The authors estimate the droplet diffusion distance as 0.63 μm for an initial droplet size of 3–6 μm, which undergoes solvent evaporation to reach diameters as small as 2.7 μm.^12^ The study concluded that the reaction on the gas–liquid interface of the microdroplets occurred faster than the bulk.

Here, we investigate the effects on reaction rates by further increasing the gas–liquid interface of the microdroplet with the addition of nanobubbles. Nonreactive N_2_ nanobubbles are introduced to increase interfacial surface areas, while CO_2_ NBs both deliver a reagent and increase interface surface areas. This approach allows evaluation of increased CO_2_ reagent use independently of potential catalytic effects produced by the nanobubble addition. CO_2_ or N_2_ NBs were generated either in 95% ACN or 5% water or both fractions as indicated in Figure 3 by yellow highlighting. We investigated three distinct NB generation methods, as our prior studies demonstrated that the mode of generation influences the extent of protein unfolding,^55^ signal enhancement, and reactivity.^58^ The NBs were made using pressure cycling (PC), flow regime switching using the Tesla valve (TV), or sonication (sonic) as described in the Materials and Methods section.

**Figure 3:**
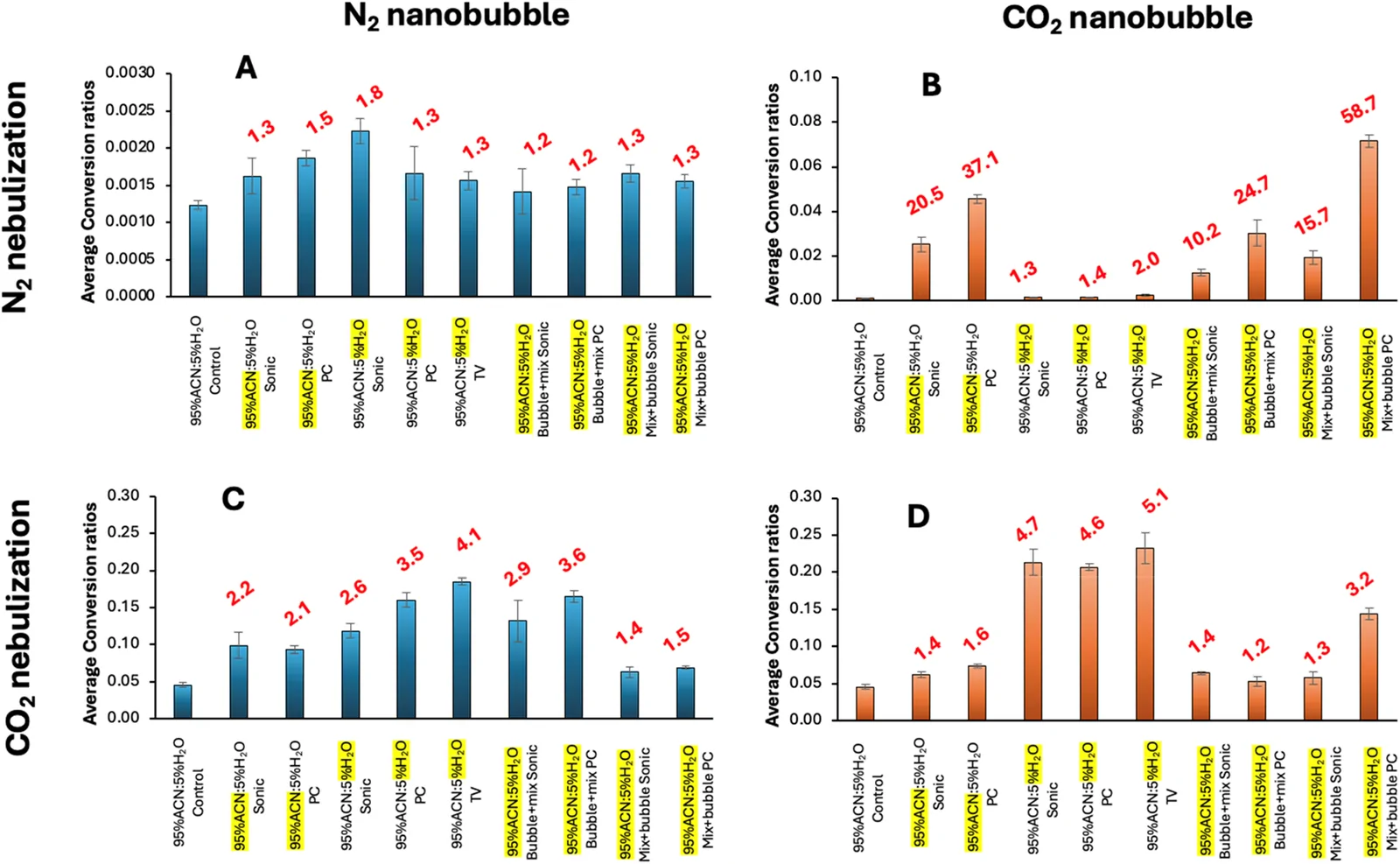
Conversion ratio of the reaction between DBPA and CO_2_ in ESI-MS using N_2_ NB (blue bars; A, C) and CO_2_ NB (orange bars; B, D) with N_2_ nebulization (A, B) or CO_2_ nebulization (C, D). NBs are added either in acetonitrile or water or both solvent fractions as indicated by the yellow highlight. Red numbers above bars indicate the acceleration factors obtained using microdroplets with added NBs compared to the control microdroplet reactions without NBs (the first bar in each panel).

The nebulization gas was also interchanged between N_2_ and CO_2_. During the preparation of N_2_ nanobubble solutions, the solvent is sparged and saturated with N_2_ gas, effectively removing dissolved CO_2_. In this control experiment, the only CO_2_ source is atmospheric diffusion onto and into the droplet after nebulization. These results of the reaction of DBPA with CO_2_ delivered as nanobubbles in microdroplets with N_2_ and CO_2_ nebulization are shown in Figure 3.

### Nitrogen Nebulization

Figure 3 A shows that the addition of **N_2_ NBs** to the solvent fractions, along with N_2_ nebulization, resulted in an acceleration factor of 1.8, with sonication producing NBs present in the 5% water fraction (Figure 3A). The very small conversion ratios observed here when DPBA is electrosprayed with N_2_ nebulization are a consequence of the low CO_2_ levels in these experiments. The acceleration factor (indicated by red numbers on top of each bar) refers to the acceleration relative to the microdroplet experiments without the NB presence, the first column of each panel.

Considering the assertion of relatively slow CO_2_ diffusion into the bulk droplet, this could imply that the reaction, occurring near or at the droplet liquid–air interface, is likely improved by NB-mediated transport of the amine reagent to the microdroplet surface. NBs possess a negative surface charge and, therefore, should experience a strong attraction to the positively charged droplet surface. This could compensate for the kinetic limitation, previously pointed out by Rovelli et al., that for reactions to be accelerated at the surface, the analyte must diffuse from the droplet interior to the interface within the droplet lifetime, and equilibrium surface coverage was shown to be reached only after 40 ms, significantly longer than the droplet lifetime of 10–20 ms.^69^

Alternatively, the NBs may create additional internal interfacial area where both CO_2_ and amine could concentrate and react. This latter scenario is more likely in the case where **CO**_**2**_ **NBs** are present, as shown in Figure 3B, and is supported by the observation that the larger NB-containing solvent fractions show a larger acceleration factor in Figure 3B.

Reagent delivery by the addition of **CO_2_NBs** in the solvent system resulted in acceleration factors between 2 and nearly 60 for NB systems, depending on the method of generation and the fraction of solvent containing the NB (Figure 3B). The greater acceleration factors observed with CO_2_ NB addition are due to increases in both the interfacial area within microdroplets and reagent availability. CO_2_ NBs in the 5% water fraction of the solvent system only resulted in an acceleration factor of 1.3–2, likely due to less CO_2_ availability (only 5% water-containing reagent). The highest acceleration factor due to the NB presence of 58 was achieved with N_2_ nebulization and CO_2_ NBs generated in the premixed solvent. Greater acceleration factors with CO_2_ NBs added in the ACN solvent fraction or the total fractions may be attributed to the 10× greater solubility of CO_2_ in ACN (0.27 M) compared to water (0.033 M).^70^ Additionally, NBs in pure organic solvents act as gas sinks,^71^ thereby leading to less stable NBs and thus the release of the gaseous reactant into solution for reaction. Alternatively, NBs may aid in the formation of secondary droplets during the fission at the Reighleigh limit of the primary microdroplet, increasing the reactive surface areas indirectly.

Near-equal conversion ratios result when carbon dioxide NBs, produced in ACN, are present (Figure 3B) with N_2_ nebulization, and for the experiment where CO_2_ nebulization is performed without the presence of NBs (Figure 3C). This demonstrates that it is equally beneficial for accelerating the conversion rate to add the CO_2_ reagent as NBs in solution as to use the CO_2_ reagent for nebulization.

The benefit of CO_2_ delivery in solution as NBs over the use of CO_2_ nebulizing gas is the significantly higher efficiency of CO_2_ utility compared to the large excess that would be used for nebulization, most of which is lost to the atmosphere.

### Carbon Dioxide Nebulization

The microdroplet reaction, upon switching the nebulization gas from N_2_ to CO_2_, resulted in overall greater conversion ratios as reported earlier.^12^ This can be seen by comparing the first bar in Figure 3A,3C. The addition of **N_2_NBs** to the solvent system resulted in further improvement of the microdroplet reactions, resulting in NB-induced acceleration factors of 2–4, depending on the method of generation and the solvent fraction used for NB preparation (Figure 3C). An acceleration factor of 4 compared with the control microdroplet reaction without bubbles was obtained when N_2_ NBs were generated by TV in the 5% H_2_O fraction.

The synergistic reagent delivery by **CO**_**2**_ **nanobubbles** and CO_2_ nebulization provided an acceleration factor of 5, and the highest overall conversion ratios, approaching 25%, when the CO_2_ NBs are created in the water fraction (Figure 3D). This is only 1.2× the conversion ratio when N_2_ NBs prepared in the water fraction by TV are used (Figure 3C), further demonstrating the catalytic or physical effects of NB presence in a system already containing excess CO_2_ from nebulization.

### Nanobubble Stability Effects

Pressure cycling generally produces less stable nanobubbles, as evidenced by their lower ζ-potentials (Table S1), increasing the availability of reagent gas and thus conversion ratios when this preparation method is used. Solutions where CO_2_ NBs are prepared by pressure cycling therefore show higher conversion ratios when the bubbles are prepared in solutions containing all or mostly ACN. A case in point is the slightly reduced conversion ratio of 3% for CO_2_ NBs produced by sonication in ACN compared to the 5% obtained for PC, both with N_2_ nebulization. When the only source for CO_2_ is the NB (Figure 3B), but the NB is very stable, the reaction proceeds slowly because it is starved of its CO_2_ reagent sequestered in the NB. On the other hand, when plenty of CO_2_ is available due to CO_2_ nebulization (Figure 3C), the reaction is accelerated because of the physical or catalytic effects of the stable NBs. This would be the case if very stable NBs result when they are produced in the water solvent. On the other hand, with N_2_ nebulization, when CO_2_ NBs are made in the total or ACN solvents, either more residual CO_2_ is still available in the solvent, or the NBs are less stable and can easily provide the reagent, leading to the large improvement factors reported in Figure 3B when produced in ACN (37×) or the total fraction (58×). The highest CR obtained with N_2_ nebulization and CO_2_ delivered as nanobubbles (7.2 %) is still greater by an acceleration factor of 1.6 compared to CO_2_ delivery by nebulization gas (4.5 %), clearly demonstrating the potential of NBs to further accelerate microdroplet reactions.

### Nanobubble versus Microdroplet Acceleration Factors

Although several orders of magnitude acceleration is reported in literature for microdroplet reactions, the exact underlying mechanism is not completely understood, and careful consideration must be given before attributing the acceleration to specific physicochemical processes, such as solvent evaporation leading to increased reagent concentration or interfacial effects governed by adsorption kinetics.^69^ Reagent diffusion and enrichment at the gas–liquid interface, as well as reagent orientation, may further contribute to reaction acceleration, as previously observed in other microdroplet reactions.^72,73^ While the acceleration factors are much higher when going from bulk to microdroplets compared to creating nanobubbles in bulk reaction solvents, this can easily be explained by the nearly 5 orders of magnitude difference in additional surface areas created by nebulization, as opposed to NB inclusion. For 1 mL of solvent nebulized as 5 μm droplets, the surface area increases to 1.2 m^2^, while adding 100 nm nanobubbles to a concentration of 1 E9 bubbles/mL would only add 0.314 cm^2^ additional surface area. For calculations, see the Supporting Information.

It is well reported that the outer convex interface of microdroplets exhibits large electric fields in the magnitude of 10^9^ V/m.^20,23^ The field is considered strong enough to oxidize OH^–^ to OH^•^ radicals, which subsequently recombine to form H_2_O_2_. The interfacial reactivity in aqueous microdroplets is curvature-dependent, as increasing the curvature (decreasing droplet radius) amplifies electric fields, thereby enhancing reaction rates.^74^ Notably, the efficiency of H_2_O_2_ generation increases with decreasing droplet size, demonstrating that enhanced interfacial curvature (1/*R*) amplifies surface charge density and electric field intensity, thereby lowering the energy barrier for an interfacial redox process and promoting otherwise unfavorable reactions.^30,75^ Nanobubbles, on the other hand, have electric fields of ∼10^6^ to 10^8^ V/m, and the interfacial geometry at the gas–water interface because the nanobubble is concave. Still, the production of OH^•^ radicals has often been described.^76,77^

Partial solvation of the reagent near the surface of both microdroplets and nanobubbles is another likely factor contributing to the observed enhancement. Molecular dynamics simulations reveal that interfacial curvature alters the H-bonding network by increasing the fraction of dangling OH groups and altering interfacial mobility. However, curvature has only a minor effect on ion solvation, and it may not by itself fully explain the enhanced reactivity in aqueous microdroplets (or nanobubbles).^78^

Another important distinction between droplets and nanobubbles is the pressure of the gas phase across the interface. In the case of droplets, the ambient pressure approximates the atmospheric pressure, while the pressure inside 200 nm NBs has been estimated to be around 30 atm due to surface tension, calculated by the Young–Laplace equation.^40,79^ This should lead to high concentrations of the gaseous reagent present at the liquid–gas interface in the case of CO_2_ NBs. The high internal pressure of NBs may provide not only a locally high concentration of CO_2_ but also an increased frequency of molecular collisions.

Although nanobubbles clearly improve reaction rates in bulk solution and further accelerate the microdroplet reactions, it is difficult to determine which specific physicochemical factor is predominantly responsible for the improvements. Plausible contributions include the curvature-associated high electric field, increased interfacial areas, and nanoscale confinement of reagents at the interfaces. In the reaction involving amines, additional factors, such as acidity/basicity, may also influence reactivity. These effects likely work together and require further investigation to understand them fully. Regardless of the origins, the simple introduction of NBs improves reaction efficiency in both bulk and microdroplet environments, demonstrating their practical utility.

## Conclusion

Nanobubbles accelerate reactions in bulk solutions and electrosprayed microdroplets. Our findings provide evidence that NBs enhance reaction kinetics by acting as catalysts and as reagent delivery agents. Several factors may contribute to the observed NB-induced acceleration of reactions, including enhanced gas solubilization and delivery in solution, increasing the gas–liquid interface by creating additional gas–liquid interfaces, and reduced diffusion distances, thereby promoting interfacial reactions. The dominating mechanism of improvement is condition-dependent. Factors such as reagent concentrations determine whether interfacial effects or reagent delivery and transport effects dominate.

The effects of NB addition were demonstrated for the Katritzky and CO_2_ sequestration reactions. In the Katritzky reaction, the NB addition to bulk solutions provided an acceleration factor of 2, while the presence of NBs in the spray solution further increased the reaction rate over that of microdroplets by an acceleration factor of 6. While the magnitude of acceleration in bulk with NB is modest compared to reported microdroplet systems, achieving enhanced conversion directly for bulk solution reactions eliminates challenges associated with droplet collection and product isolation, thereby facilitating scalability and practical implementation in large reactors.

For the CO_2_ sequestration reaction, nearly 60-fold improvement for NB systems was obtained, without excessive CO_2_ addition as nebulizing gas, delivering a much more efficient reaction, considering CO_2_ inputs. NB-assisted reaction acceleration in bulk also has the advantage over microdroplets for ease of product collection and simplifying potential future scaling-up efforts. The results demonstrate the potential of NBs as agents for reaction rate enhancement in synthetic and analytical chemistry.

## Supplementary Material



## Notes

Physical characterization of the nanobubbles; mass spectra of the Katritzky reaction at various time intervals with and without nanobubbles; ion intensities used to calculate conversion ratios in Figure 1; optimization of water fraction in the reaction between CO_2_ and DBPA; optimization of the NB-containing water fractions; conversion ratios for the reaction with DBPA and micromolar concentrations of water; and calculations for the increases in interfacial areas with nebulization compared to nanobubble inclusion (PDF)
