# Supplementary material for: Nanobubbles in Bulk Solution and Electrosprayed Microdroplets Accelerate Chemical Reactions
Source: J Am Soc Mass Spectrom. 2026 Sep 9;37(10):2609–17. doi: 10.1021/jasms.6c00359 (PMC13651626; doi:10.1021/jasms.6c00359)
Supplement: Supplementary file 1 [file js6c00359_si_001.pdf]

Supplemental Information

Nanobubbles in bulk solution and electrosprayed microdroplets accelerate chemical reactions

Bincy Binny, George Joseph, Andre R Venter*

Western Michigan University, Kalamazoo, Michigan, 49008-5413
andre.venter@wmich.edu

Table of contents

		page
**Table S1**	Size, concentration and zeta potential of CO_2 and N_2 nanobubble in water.	S-2
**Figure S1**	Mass spectrum of Katritzky reaction at various time intervals with and without nanobubble addition.	S-3
**Figure S2**	Product and reagent ion intensities for the Katritzky reaction used to calculate conversion ratios in Figure 1.	S-4
**Optimization of water fraction in the reaction between CO_2 and DBPA**		S-5
**Figure S3**	Optimizing NB containing water fraction of the solvent system.	S-6
**Figure S4**	99% ACN and 95% ACN solvent system optimization.	S-7
**Figure S5**	Conversion ratios for reaction with DBPA and CO_2 with micromolar water concentrations.	S-7
**Calculation of surface area increases for nebulized solvents inclusion**		S-8
**Calculation of surface area increases with inclusion of nanobubbles**		S-9

Table S1: Size, concentration and zeta potential of CO_2 and N_2 nanobubbles in H_2O measured by using a Nanoparticle Tracking Analysis system (NTA).

	**CO_2 nanobubbles**			**N_2 nanobubbles**		
**Generation method**	Diameter (nm)	NBs/ml	Zeta potential (mV)	Diameter (nm)	NBs/ml	Zeta potential (mV)
Sonication	70	3.1 E+08	-36.1	73	4.3 E+08	-40.7
Pressure Cycling	81	1.1 E+09	-31.9	89	1.9 E+09	-37.3
Tesla Valve	67	3.3 E+08	-37.6	71	5.5 E+08	-43.2

Figure S1: Mass spectrum of Katritzky reaction from time 0 min to 60 mins
using control (left panel) and nanobubbles (right panel).
A) full range showing both reagent (yellow bar) and product (orange bar)
B) Zoomed in spectra for the product ions

A)

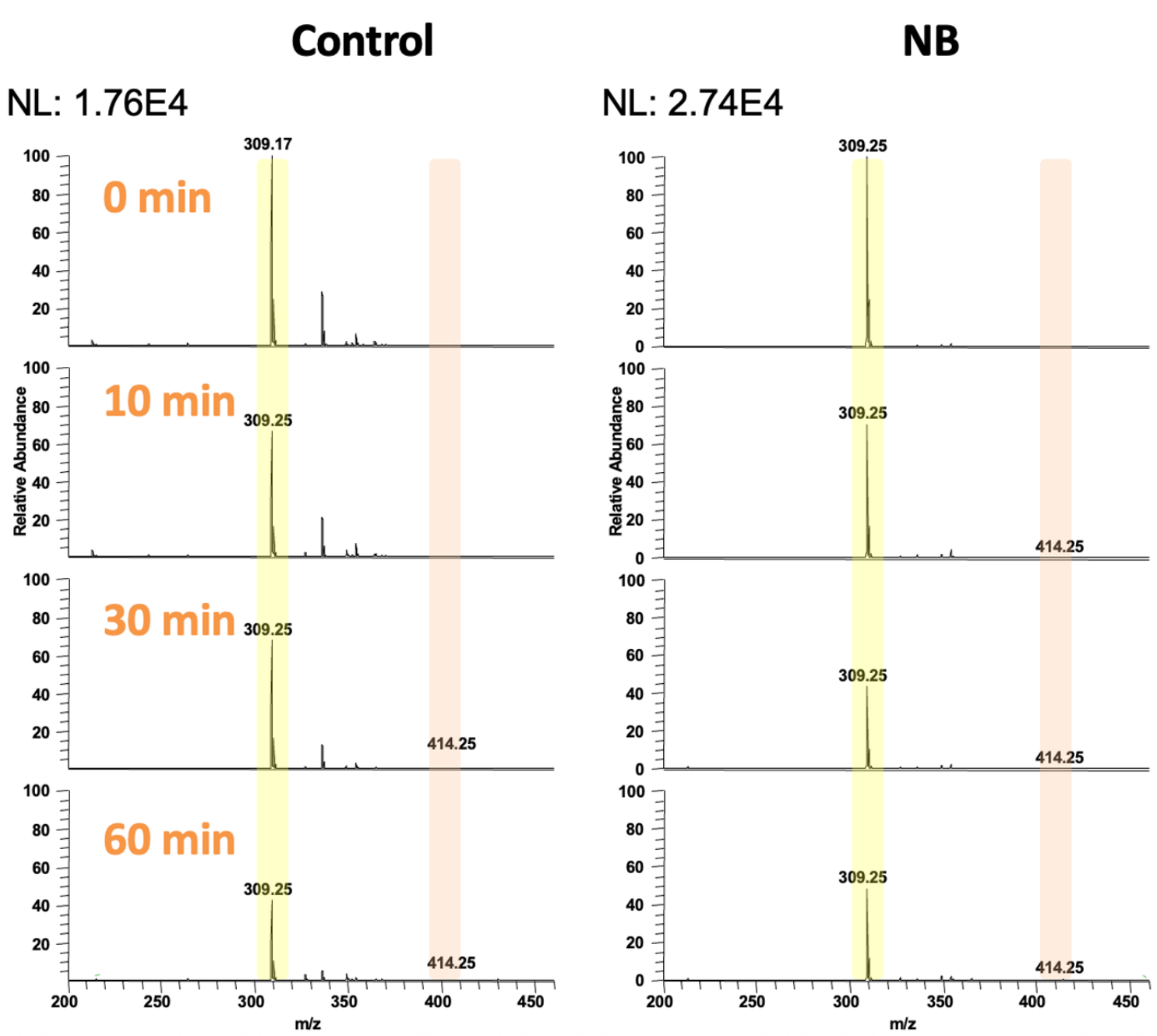

B)

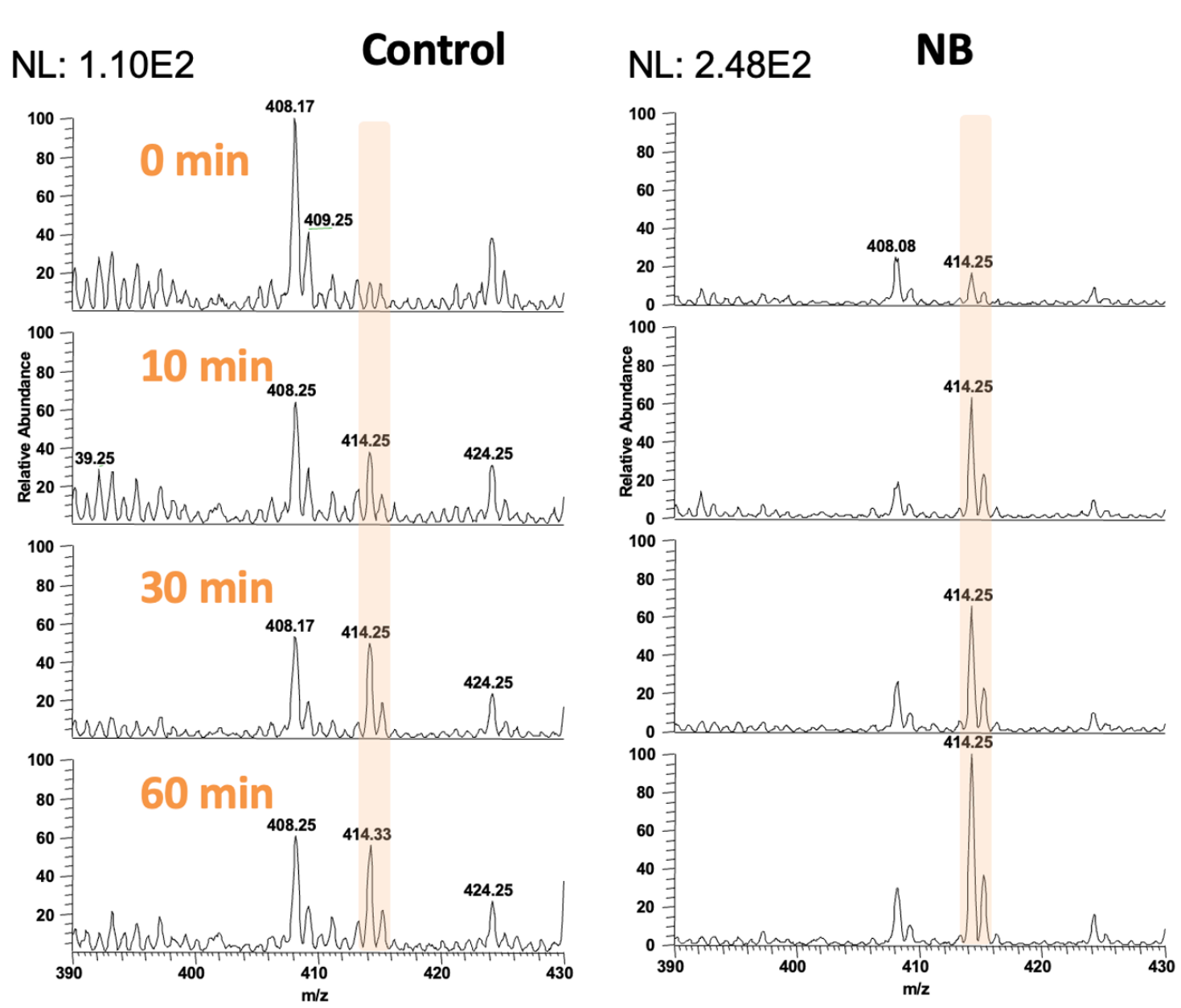

Figure S2: Intensity of (A) reagent and (B) product of Katritzky reaction over time.

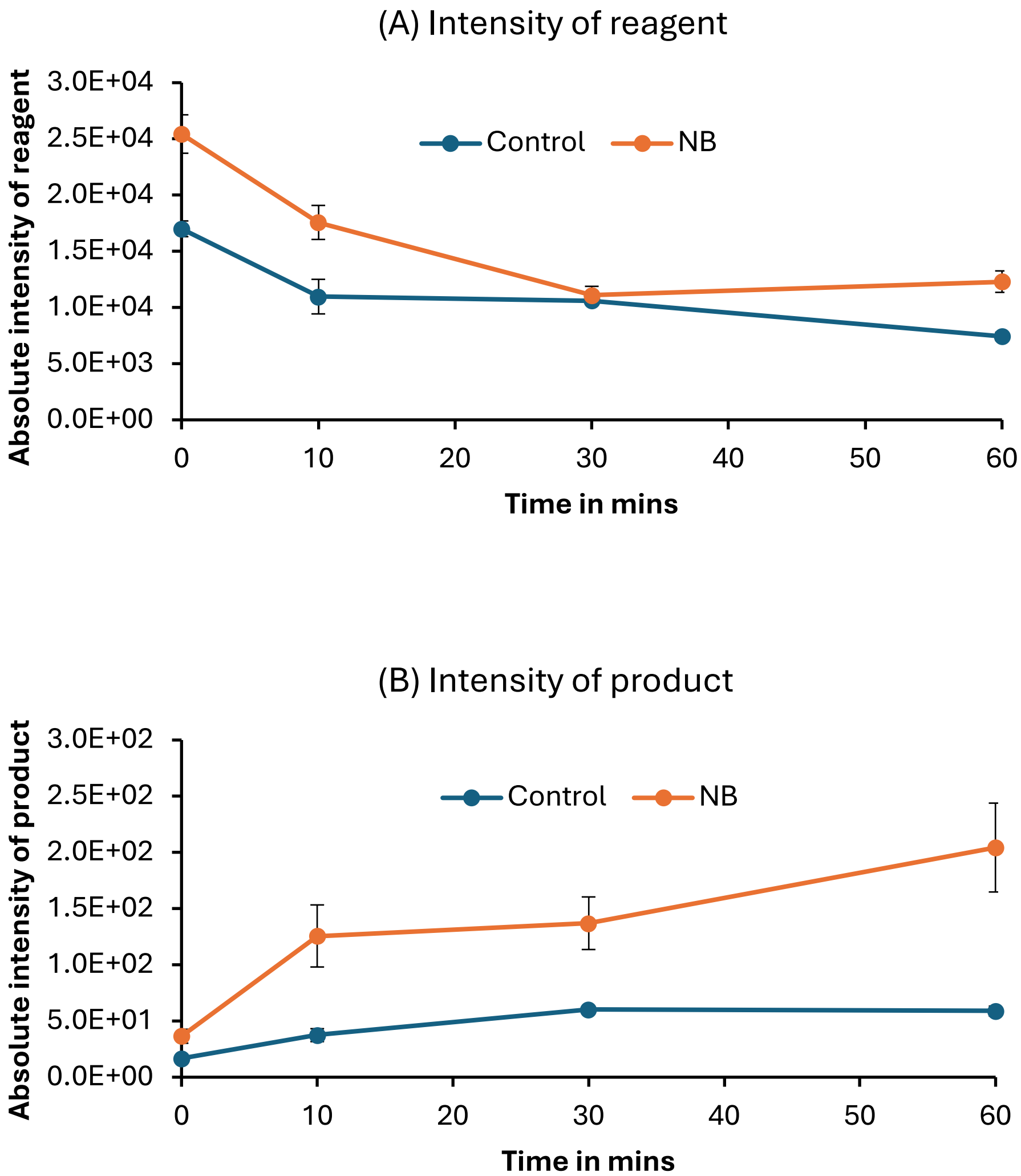

Optimization of water fraction in the reaction between CO_2 and DBPA

Prior work on the reaction between CO_2 and DBPA optimized the water fraction to 5% of the total solvent composition.[1] The highest conversion ratios obtained when CO_2 NBs (Figure 3D in main text) were present in this small fraction of water prompted further investigation into the potential role of increasing the total number of NBs in the system by increasing the fraction of the NB containing aqueous phase. The solvent optimization for the reaction is depicted in Figure S3. Interestingly, consistent with the earlier findings, smaller amounts of water (~5%) dramatically enhanced conversion ratios. In all cases NB addition showed greater acceleration factors, but the highest NB induced acceleration factor of 5X was obtained with the lowest concentration of water compared to around 2X for the other cases.

Since 5% water was the smallest concentration studied previously,[1] we investigated even lower concentrations of water, shown in Figure S4. Reducing the aqueous fraction further to 1% the conversion ratio for the control system without NBs increased even further relative to the 5% water containing control (Figure S4). NB addition in water fraction gave highest conversion ratios for both 5% H_2O and 1% H_2O systems. CO_2 NB addition in the 1% H_2O produced a conversion ratio almost as good as when produced in 5% H_2O. The enhancement factor is however lower due to small number of NBs, which scales with the smaller water fraction. Lowering water concentration to equimolar concentration of DBPA resulted in the highest CR (Figure S5).

[1] Huang, K.-H.; Wei, Z.; Cooks, R. G. Accelerated reactions of amines with carbon dioxide driven by superacid at the microdroplet interface. *Chemical Science* **2021**, *12* (6), 2242-2250, 10.1039/D0SC05625A

Figure S3. Optimization of NB containing water fraction for the reaction between the amine DBPA and CO_2. CO_2 NBs were generated by the three different generation methods in the aqueous fraction of the solvent system before mixing.

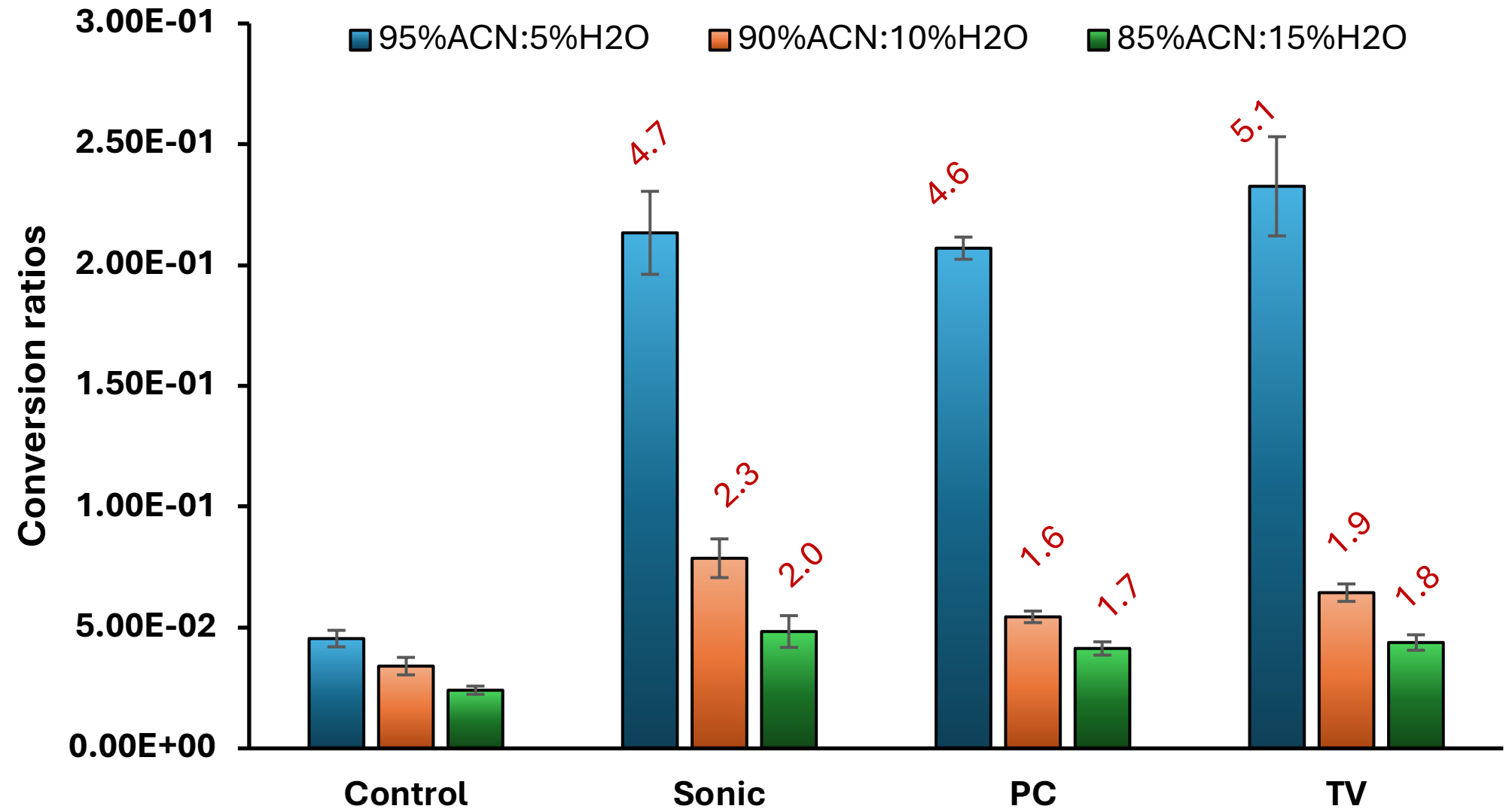

Figure S4: Optimizing solvent fractions containing NBs using 95%ACN: 5%H_2O (blue bars) and 99%ACN: 1% H_2O (orange bars) solvent systems. Yellow highlighted letters indicate the fraction were in CO_2 NBs were created. Red letters indicate the conversion ratio enhancement factors. Addition of NB in the total fraction was performed in two ways: NBs were created in separate solvents followed by mixing; or the solvents were blended before preparing NBs.

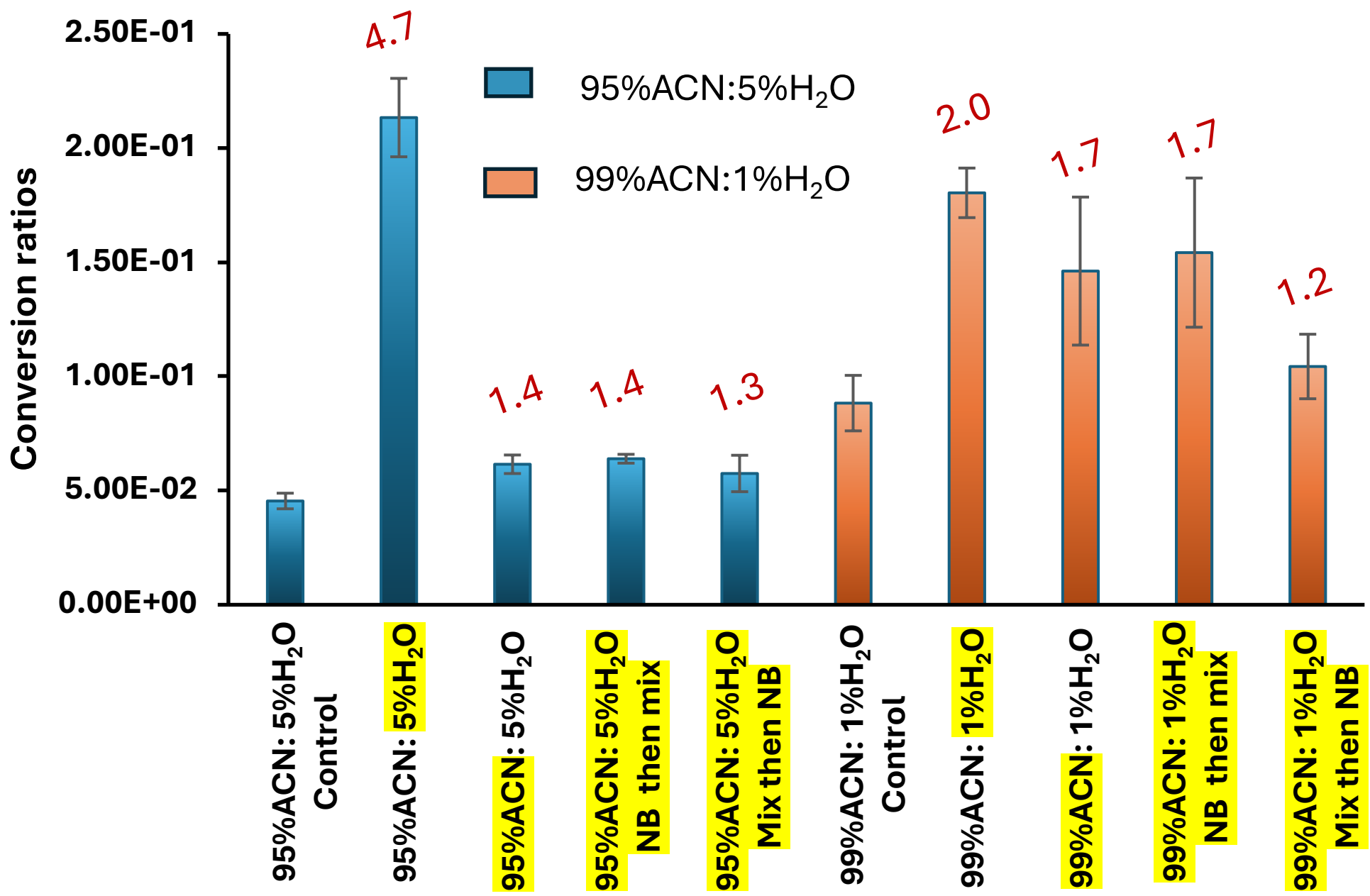

Figure S5: 10 µM DBPA in ACN with varying concentrations of water using CO_2 nebulization.

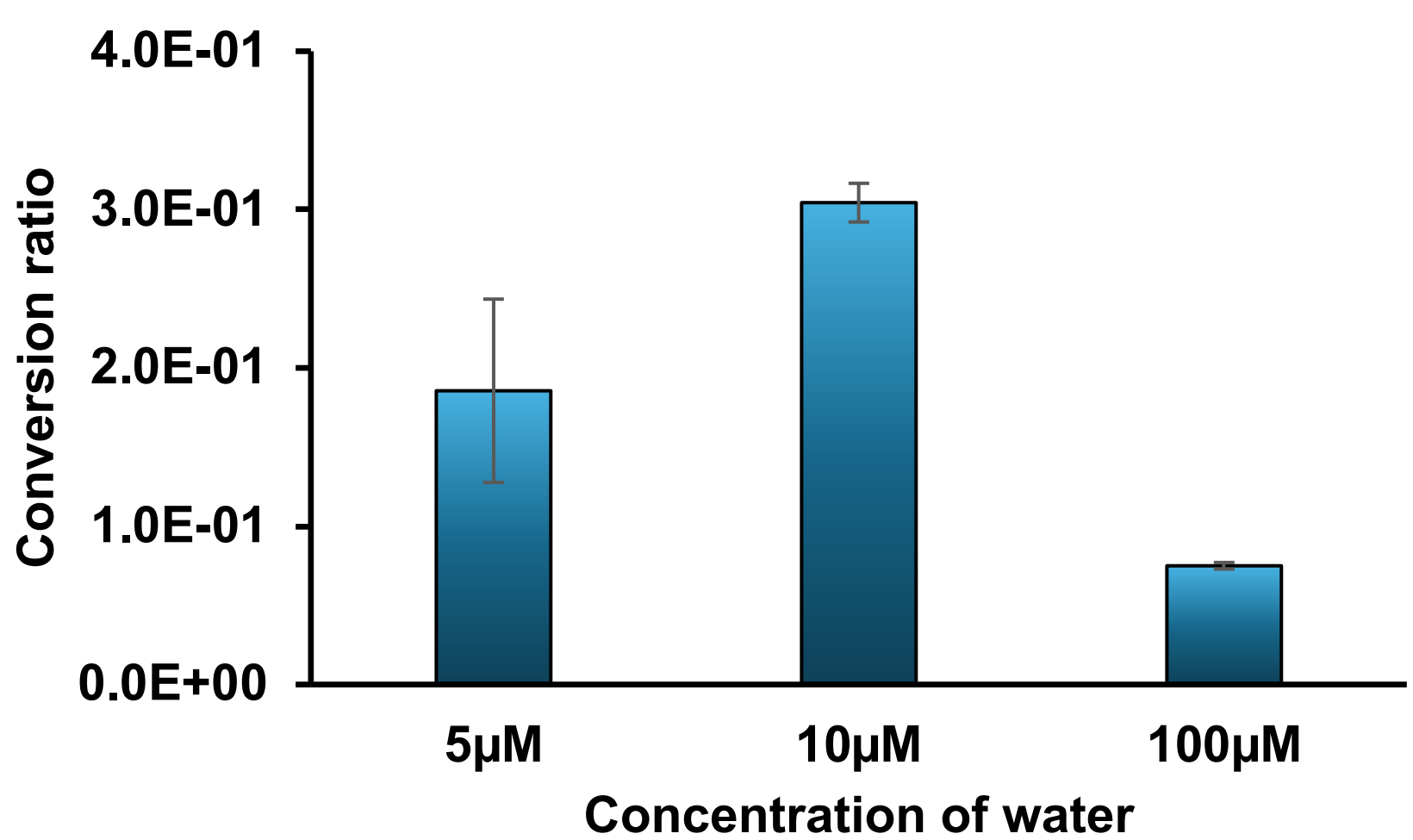

Calculation of surface area increases for nebulized solvents and nanobubble inclusion.

Nebulization of 1 mL solvent as 5 µm droplets:

1. **Calculations of individual droplet properties:**

   - **Volume of one droplet (v):**

$$v = \frac{4}{3}\pi(2.5 \times 10^{-6})^3 \approx 6.545 \times 10^{-17} \text{ m}^3$$

   - **Surface area of one droplet (a):**

$$a = 4\pi r^2 = 4\pi\,(2.5 \times 10^{-6})^2 \approx 7.854 \times 10^{-11} \text{ m}^2$$

2. **Total number of droplets (N):**

$$N = \frac{Total\ volume}{Droplet\ volume} = \frac{1 \times 10^{-6} m^3}{6.545 \times 10^{-17} m^3} \approx 1.528 \times 10^{10}\ droplets$$

3. **Calculations of total surface area (A_{total})**

The relationship between total volume, diameter, and total surface area for spherical drops:

$$A_{total} = N \times a = \frac{6V}{d}$$

Plugging in the values:

$$A_{total} = \frac{6 \times 10^{-6}\ m^3}{5 \times 10^{-6}\ m} = \mathbf{1.2\ m^2}$$

Inclusion of 1.0 x10^9 nanobubbles with D = 100 nm

1. **Surface area of one bubble (A_{single})**

   Assuming the bubbles are perfectly spherical, the formula for the surface area of a sphere is:

$$A_{single} = 4\pi r^2 \ = \ \pi d^2$$

- In square centimeter (cm^2)

$$A_{single} = \pi \times (1 \times 10^{-5}\, cm)^2 = \pi \times 10^{-10}\, cm^2 \approx 3.1416 \times 10^{-10}\ \mathrm{cm}^2$$

- In square meter (cm^2)

$$A_{single} = \pi \times (1 \times 10^{-7}\, m)^2 = \pi \times 10^{-14}\, m^2 \approx 3.1416 \times 10^{-14}\ \mathrm{m}^2$$

2. **Total interfacial surface area per mL:**

Multiply the surface area of a single bubbles by the total number of bubbles per milliliter:

$$A_{total} = N \times A_{single}$$

- In square centimeter (cm^2/mL):

$$A_{total} = 10^9 \times (\pi \times 10^{-10}\, cm^2) = 0.1\pi cm^2 \approx \mathbf{0.3142\ cm^2}$$

- In square meter (m^2/mL)

$$A_{total} = 10^9 \times (\pi \times 10^{-14}\, m^2) = 10^{-5}\pi m^2 \approx 3.1416 \times 10^{-5}\ \mathrm{m}^2$$
